# Chronic otitis media with effusion in children with adenoidal hypertrophy: Development of a diagnostic prediction model

**DOI:** 10.17305/bb.2025.12825

**Published:** 2025-11-19

**Authors:** Yu Wu, Yuan Jiang, Yan-Xun Han, Da-Ming Wang, Chang-Yu Yao, Ye-Hai Liu

**Affiliations:** 1 Department of Otorhinolaryngology, Head and Neck Surgery, The First Affiliated Hospital of Anhui Medical University, Hefei, China; 2 Department of Otorhinolaryngology, Head and Neck Surgery, The People’s Hospital of Chizhou, Chizhou, China

**Keywords:** Adenoid hypertrophy, children, chronic otitis media with effusion, nomogram, risk factor

## Abstract

Chronic otitis media with effusion (COME) is a prevalent condition that poses significant risks to the growth and development of children with adenoidal hypertrophy (AH). This study investigates the risk factors associated with COME in children diagnosed with AH and establishes a clinical prediction nomogram to enhance diagnostic accuracy. The study included 311 children with AH, diagnosed through lateral nasopharyngeal radiographs, from the Department of Otorhinolaryngology Head and Neck Surgery at the First Affiliated Hospital of Anhui Medical University. Risk factors were identified using the least absolute shrinkage and selection operator (LASSO), while Firth’s penalized logistic regression analysis was employed to further refine the variables and develop a predictive model. The model’s performance was assessed using the C-index, calibration curve, and decision curve analysis, with internal validation conducted through bootstrapping. The resulting predictive nomogram included four key risk factors: young age, vitamin D3 deficiency, degree of AH, and tympanometry results. The model exhibited strong predictive capabilities, achieving a C-index of 0.945 (95% confidence interval: 0.941, 0.949). Bootstrapping validation confirmed a high C-index of 0.934. The calibration curve demonstrated good alignment, while the decision curve indicated a net benefit across thresholds of 10%–90%. This nomogram—incorporating tympanometry, AH degree, serum vitamin D3 levels, and age—serves as a valuable tool for clinicians and families in assessing the risk of COME in children with AH.

## Introduction

Otitis media with effusion (OME) is characterized by the presence of fluid in the middle ear without acute inflammation. A duration of OME exceeding three months is classified as chronic OME (COME) [1]. COME is among the most prevalent conditions affecting early childhood. Epidemiological studies have reported incidence rates of COME in adolescents ranging from approximately 15% to 30% in the United States, 32.3% in India, 16.5% in children attending primary clinics in South Africa, and between 1.16% and 30.7% in various regions of China [1–4]. Research by Tos [5] indicated that children experience OME at least once during their early years. COME is a significant contributor to hearing loss in children and can adversely impact speech and social development [6]. Additionally, prolonged negative pressure in the middle ear may influence vestibular function, leading to varying degrees of balance disorders in children during daily activities [7].

Research has identified multiple etiological factors associated with COME, including respiratory tract infections, adenoidal hypertrophy (AH)—influenced by the degree and location of the adenoid—maxillofacial deformities, allergic and immune responses, family environment, and breastfeeding practices [8–12]. Adenoids, functioning as immune lymphoid tissues in the nasopharynx, play a crucial role in preventing upper respiratory infections during infancy. However, the lymphoid tissues of young children may undergo hyperplastic changes due to repeated exposure to various pathogens, such as bacteria and viruses, leading to mouth breathing. Prolonged mouth breathing can contribute to maxillofacial deformities. Austin demonstrated that hyperplastic adenoids can mechanically obstruct the pharyngeal ostium of the Eustachian tube, resulting in sustained negative pressure in the middle ear and tympanic cavity, a common cause of COME in young children [13, 14].

Tympanometry serves as an effective, non-invasive method for assessing middle ear pressure, although it requires a certain level of cooperation from children. However, traditional tympanometry lacks 100% accuracy in diagnosing the presence of fluid in the middle ear [15, 16], particularly in type C tympanograms at 226 Hz [11]. Myringotomy and tympanocentesis are regarded as gold standards for diagnosing middle ear effusion but also necessitate high levels of cooperation from both the child and parents. These procedures must be performed under general anesthesia in pediatric patients and carry operational risks, such as poor healing of the tympanic membrane and damage to the auditory ossicles. Consequently, there is a need for a non-invasive and accurate diagnostic method for COME that integrates tympanometry with additional clinical factors. Nomograms, as visualization tools for statistical models, can consolidate multiple clinical variables, thereby enhancing the interpretability of predictive models.

This study aims to analyze and identify the risk factors for COME in children with AH, develop a diagnostic prediction model, and present it as a nomogram. The model is designed to provide a supplementary diagnostic tool for clinical settings in primary hospitals in China while minimizing the risks associated with invasive procedures.

## Materials and methods

This study included children who exhibited persistent nocturnal mouth breathing and underwent surgical treatment between October 2021 and September 2022 at the Department of Otorhinolaryngology, Head and Neck Surgery, The First Affiliated Hospital of Anhui Medical University. All participants presented with symptoms of consistent mouth breathing and were diagnosed with AH through lateral nasopharyngeal radiographs, with or without accompanying hearing loss. Prior to surgery, all children received conservative treatment for a minimum of three months, which included intranasal steroid therapy and oral montelukast sodium, among other interventions. The children in this study showed inadequate improvement following these conservative measures. All pediatric patients underwent fasting blood sampling within 24 h of admission and, barring any contraindications, received surgical intervention within 72 h. Exclusion criteria encompassed a history of trauma to the head, middle, or inner ear; nasopharyngeal tumors; Down syndrome; mental illness; intellectual disabilities; congenital hearing impairment; significant comorbidities (e.g., heart, liver, kidney, or hematopoietic disorders); severe malnutrition; severe rickets; congenital cleft palate; craniofacial abnormalities; or developmental disorders of the nervous system. Family members of the patients provided informed consent and understanding regarding the study.

Invasive examinations often provoke resistance and distress in children, complicating the subjective assessment of AH classification. Given that lateral nasopharyngeal projection is more readily accessible in Chinese primary hospitals and more acceptable to children, this study utilized lateral nasopharyngeal projection results instead of electronic laryngoscopy to ascertain the presence of AH [17, 18]. The adenoid/nasopharyngeal (A/N) ratio served as the metric for evaluating the degree of AH. The thickness of the adenoid (A) was defined as the vertical distance from the most prominent point of the inferior margin of the adenoid to the tangent line of the occipital clivus. The width of the nasopharyngeal cavity at the most prominent part of the adenoid (N) was determined as the vertical distance from the posterior end of the hard palate to the intersection of the pterygoid plate and the skull base. The A/N ratio was calculated by dividing these two measurements. Various diagnostic methods for AH in children using lateral nasopharyngeal projection have been proposed both domestically and internationally, including the Fujioka diagnostic criteria [19]. After careful consideration of multiple standards, we selected the criterion most appropriate for the Chinese context. Based on a literature review [19–23], AH was classified into three categories: normal (A/N ≤ 0.60), moderate (0.60 < A/N ≤ 0.70), and pathological (A/N > 0.70). Two senior attending physicians independently interpreted the lateral nasopharyngeal radiographs. In cases where discrepancies in A/N ratio measurements arose, a chief physician made the final determination. Otherwise, the measurement from the more experienced senior attending physician was used. All physicians were blinded to the clinical data. The A/N ratios for the children in this study were found to be >0.60. Tympanometry was conducted using the AT235 device by Interacoustics in a quiet environment with a 226 Hz probe tone, and results were classified according to the Liden–Jerger classification system. Each patient underwent tympanometry twice: first during the initial clinic visit and again within 24 h of hospital admission. The tympanometry results obtained during hospitalization were used for data analysis. For further statistical analysis, patients were divided into three groups based on tympanometry results: Group A included children with tympanogram A in both ears (AA), Group C included children with tympanogram C (CC, AC, CA), and Group B included children with tympanogram B (BB, BC, CB, AB, BA) [24]. All participants underwent adenoidectomy and tympanocentesis under general anesthesia. If clinically indicated, these pediatric patients may subsequently receive myringotomy with or without grommet insertion. Patients were further categorized based on the presence or absence of middle ear effusion: AH with and without COME.

Relevant demographic data were extracted from medical records, including sex, age, duration of AH, height, weight, and the season of hospitalization as defined by the astronomical division method. Nutritional status was assessed using age- and sex-adjusted body mass index (BMI). According to the World Health Organization (WHO) 2006 child growth standards, subjects with a BMI below the 5th percentile for their sex and age group were classified as underweight, those with a BMI between the 85th and 95th percentiles were categorized as overweight, and those with a BMI at or above the 95th percentile were considered obese [25, 26]. Serum vitamin D3 levels were measured using a chemiluminescence immunoassay (CLIA) platform (CL-8000i, Mindray), with a coefficient of variation of ≤10%. Vitamin D3 deficiency was defined as a serum level of ≤20 ng/mL [27]. The percentages of neutrophils, lymphocytes, eosinophils, monocytes, and basophils were recorded during routine blood examinations. Additionally, allergen test results and total immunoglobulin E (IgE) levels were included.

### Ethical statement

The study was conducted in accordance with the principles of the Declaration of Helsinki and was approved by the ethics committee of The First Affiliated Hospital of Anhui Medical University (Approval No. PJ 2023-13-30). Informed consent was obtained from the parents of all participants.

### Statistical analysis

Descriptive statistics were performed using IBM SPSS Statistics for Windows, version 26.0. Continuous data with normal distributions were expressed as means ± standard deviation, while continuous data with skewed distributions were presented as medians (p25, p75), and discrete data were reported as *N* (%). Prior to data filtering, continuous variables were categorized based on the 5th, 35th, 65th, and 95th percentiles [28]. The least absolute shrinkage and selection operator (LASSO) method, suitable for high-dimensional data reduction, was employed to select optimal predictive features among the risk factors associated with AH in children [29, 30]. A Firth’s penalized logistic regression was conducted for final variable selection and predictive model development, utilizing the selected features to estimate the probability of outcome occurrence [31]. A two-sided *P* value of <0.05 was considered statistically significant. The predictive model was represented as a nomogram.

## Results

This study involved 311 children diagnosed with AH. The male-to-female ratio among participants was approximately 3:2, with 186 male and 125 female patients categorized into two groups based on tympanocentesis findings: those with COME and those without. Table 1 summarizes the demographic and clinical characteristics of the participants.

**Table 1:** Univariate analysis of potential risk factors for chronic otitis media with effusion (COME) in children with adenoid hypertrophy (AH)

**Variables**		**AH with COME (*n* ═ 66)**	**AH without COME (*n* ═ 245)**	**Total (*n* ═ 311)**	**Test statistics**	***P* value**
Age (year)		6 (5, 8)	6 (4, 8)	6 (4, 8)	*Z* ═ −1.217	0.223
Sex					χ^2^ ═ 0.022	0.881
	Male	40 (60.6%)	146 (59.6%)	186 (59.8%)		
	Female	26 (39.4%)	99 (40.4%)	125 (40.2%)		
AH course (month)		18 (9.75, 36)	18 (12, 33)	18 (12, 36)	*Z* ═ −0.11 4	0.909
Season					χ^2^ ═ 4.419	0.031
	Winter and spring	39 (59.1%)	87 (35.5%)	126 (40.5%)		
	Summer and autumn	27 (40.9%)	158 (64.5%)	185 (59.5%)		
BMI					χ^2^ ═ 2.210	0.530
	Normal	41 (62.1%)	150 (61.2%)	191 (61.4%)		
	Underweight	0 (0.0%)	7 (2.9%)	7 (2.3%)		
	Overweight	8 (12.1%)	33 (13.5%)	41 (13.2%)		
	Obesity	17 (25.8%)	55 (22.4%)	72 (23.1)		
A/N ratio		0.82 (0.76, 0.88)	0.79 (0.72, 0.86)	0.80 (0.72, 0.86)	*Z* ═ −2.257	0.024
Degree					χ^2^ ═ 2.417	0.116
	Moderate	9 (13.6%)	55 (22.4%)	64 (20.6%)		
	Pathologic	57 (86.4%)	190 (77.6%)	247 (79.4%)		
Tympanometry					χ^2^ ═ 106.857	<0.001
	Normal	2 (3.0%)	178 (72.6%)	180 (57.9%)		
	C type	24 (36.4%)	34 (13.9%)	58 (18.6%)		
	B type	40 (60.6%)	33 (13.5%)	73 (16.7%)		
VD3					χ^2^ ═ 37.851	<0.001
	Normal	23 (34.8%)	184 (75.1%)	211 (67.8%)		
	VD3 deficiency	43 (65.2%)	61 (24.9%)	100 (32.3%)		
Allergen test					χ^2^ ═ 0.017	0.896
	Normal	34 (51.5%)	124 (50.6%)	158 (50.1%)		
	Positive	32 (48.5%)	121 (49.4%)	153 (49.9)		
Total IgE		60.25 (27.80, 166.00)	56.30 (23.40, 134.00)	56.40 (25.30, 135.00)	*Z* ═ −0.500	0.617
NEUT%		44.90 (37.15, 53.15)	43.80 (35.10, 51.50)	44.00 (36.00, 52.20)	*Z* ═ −1.406	0.160
LY%		43.29±9.91	45.46±11.46	45.00±11.17	*t* ═ 1.403	0.162
MONO%		5.90 (4.90, 7.03)	6.10 (5.10, 6.90)	6.0 (5.10, 7.00)	*Z* ═ −0.029	0.977
EOS%		2.95 (1.80, 5.90)	3.10 (2.10, 5.35)	3.10 (2.00, 5.40)	*Z* ═ −0.731	0.465
BASO%		0.50 (0.30, 0.80)	0.50 (0.40, 0.70)	0.50 (0.40, 0.70)	*Z* ═ −0.09 2	0.927

Abbreviations: BASO%: Percentage of basophils in routine blood examinations; BMI: Body mass index; EOS%: Percentage of eosinophils in routine blood examinations; LY%: Percentage of lymphocytes in routine blood examinations; MONO%: Percentage of monocytes in routine blood examinations; NEUT%: Percentage of neutrophils in routine blood examinations; Season: Season of hospitalization.

**Figure 1.**
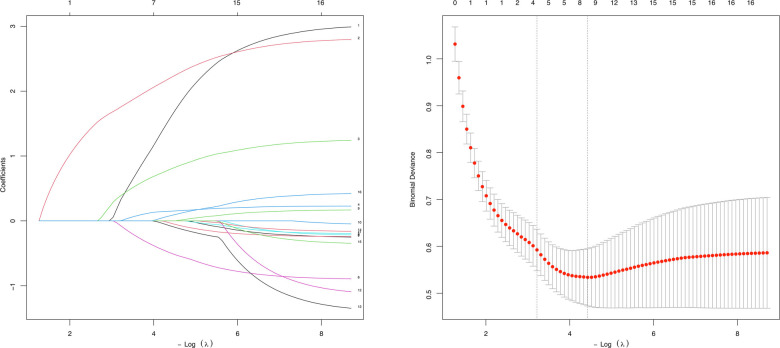
**Factor selection utilizing LASSO regression analysis.** LASSO regression in 311 children with adenoidal hypertrophy produced coefficient profiles for all candidate variables across log(λ) (left) and the cross-validated deviance curve (right). At the optimal value of λ, eight predictors had non-zero coefficients: age, preoperative tympanometry results, vitamin D3 deficiency, A/N ratio, degree of adenoidal hypertrophy, season of hospitalization, percentage of lymphocytes, and percentage of basophils. Abbreviation: LASSO: Least absolute shrinkage and selection operator.

**Figure 2.**
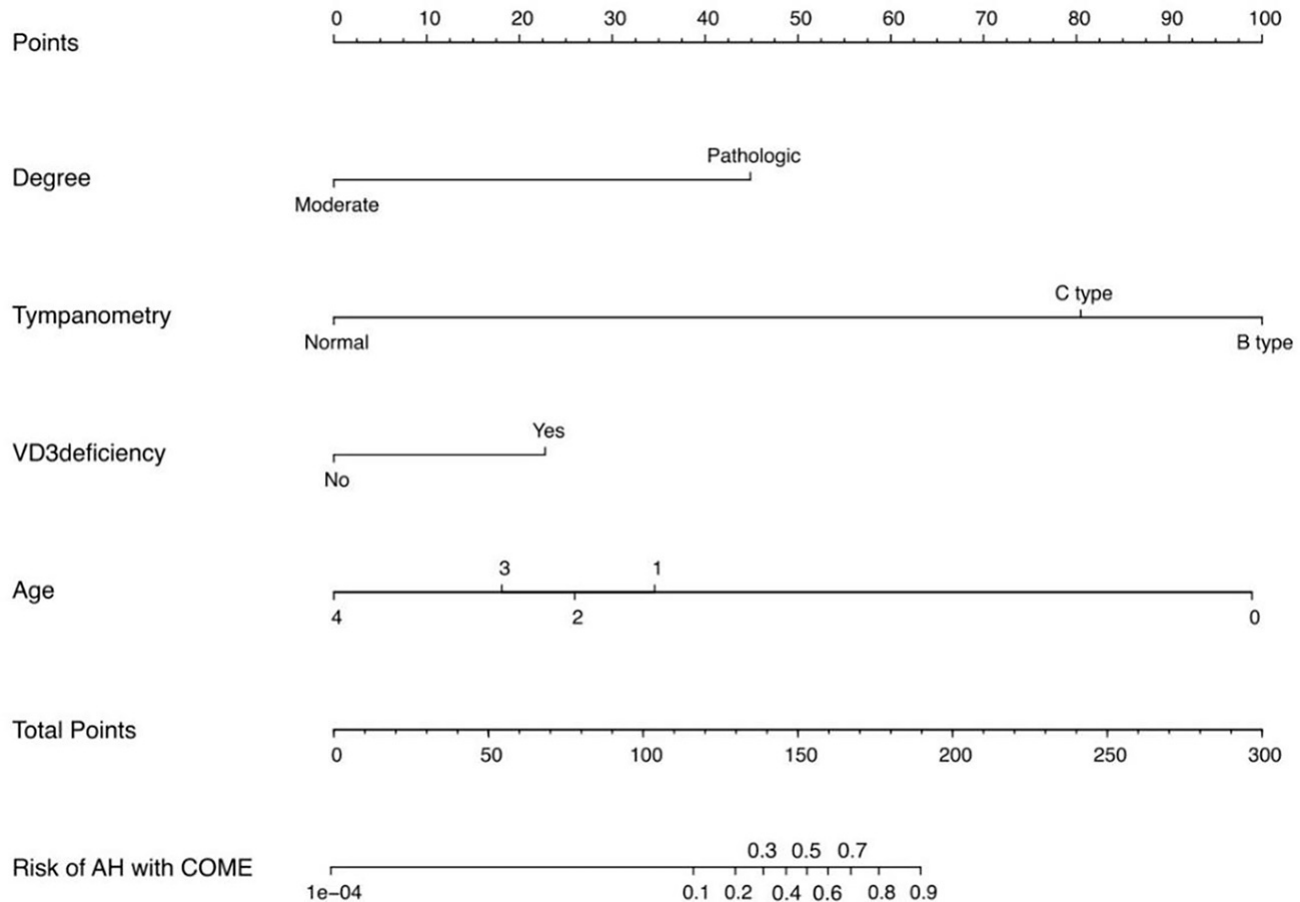
**Nomogram for predicting adenoid hypertrophy (AH) in patients with chronic otitis media with effusion (COME).** This nomogram is constructed using variables including age, tympanometry results, vitamin D3 deficiency, and the degree of adenoid hypertrophy. The age variable is encoded as follows: 0 indicates ≤3 years; 1 indicates >3 to ≤5 years; 2 indicates >5 to ≤7 years; 3 indicates >7 to ≤11 years; and 4 indicates >11 years.

**Figure 3.**
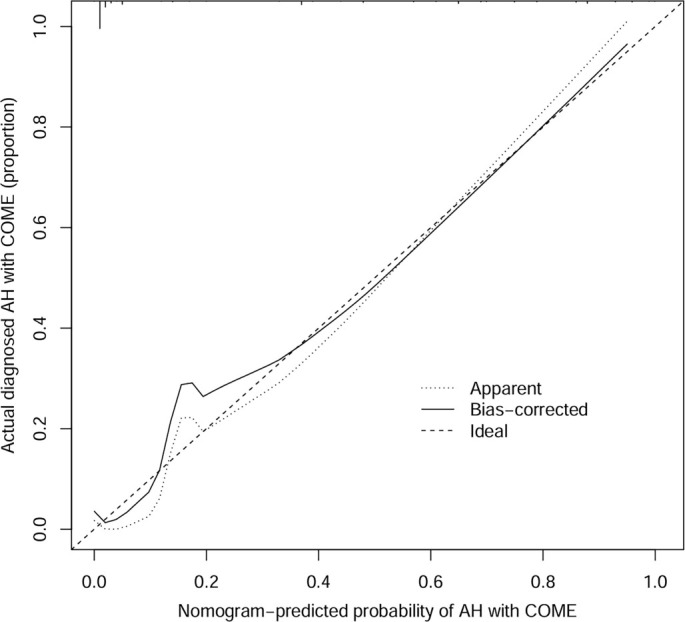
**Calibration curves of the nomogram.** The bias-corrected line closely aligns with the ideal line, indicating a strong consistency between the model’s predicted probabilities and the observed outcomes. Abbreviations: AH: Adenoid hypertrophy; COME: Chronic otitis media with effusion.

**Figure 4.**
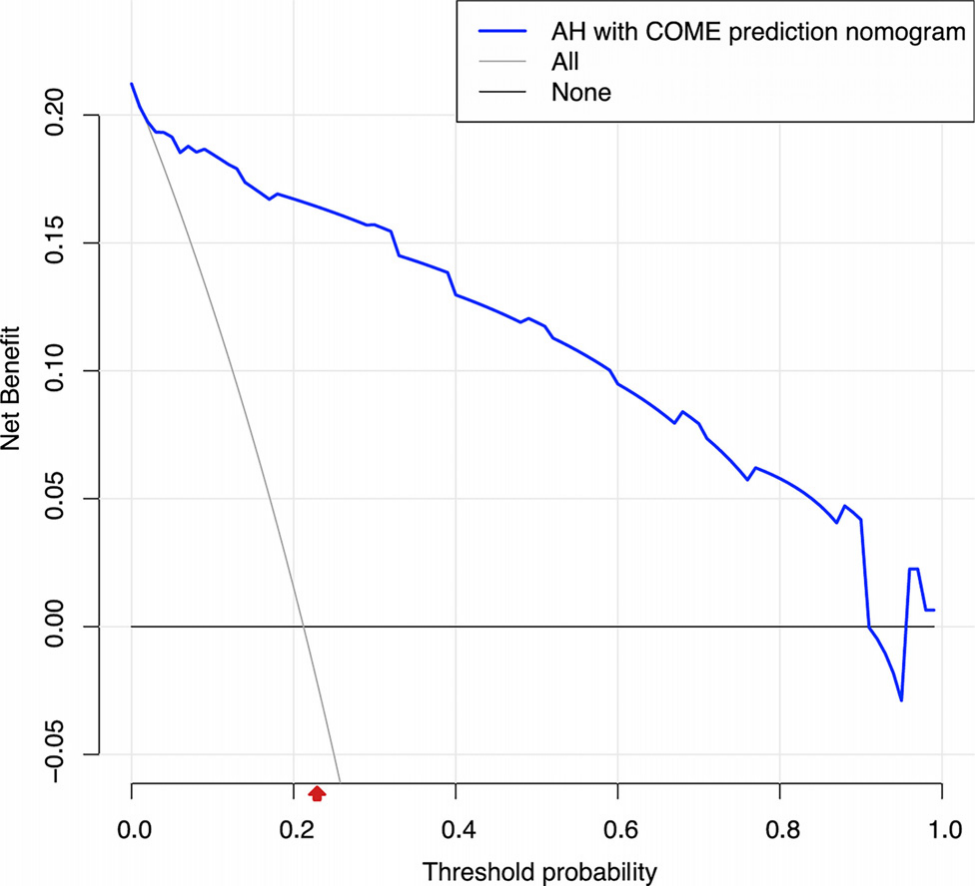
**Decision curve analysis of the prediction nomogram of AH with COME.** This curve is based on the predicted probabilities derived from the apparent model, represented by the blue line. The figure compares the net benefits of three strategies: (1) the predictive nomogram; (2) the “All” strategy (Treat All), where all children with AH receive routine intervention; and (3) the “None” strategy (Treat None), where no additional intervention is administered to any child. The results indicate that across a broad threshold probability range of 10% to 90%, utilizing our model for clinical decision-making provides a higher net benefit compared to both the “All” and “None” strategies. Across this range of threshold probabilities (10% to 90%), the model provides clinical value by effectively identifying high-risk patients for intervention at any chosen threshold, while avoiding overtreatment of low-risk individuals. The red arrow denotes the incidence rate (21.2%) of COME in children with AH as observed in this study. Abbreviations: AH: Adenoid hypertrophy; COME: Chronic otitis media with effusion.

To address the limitations of linear assumptions, this study opted not to construct a full restricted cubic spline (RCS) model. Instead, we utilized four recommended knot positions (p5, p35, p65, p95) to convert continuous variables into multi-categorical variables (Table 2), thereby simplifying the analytical process while still capturing potential nonlinear trends [28]. Employing LASSO regression, we identified eight key features from the 311 children: age, preoperative tympanometry results, vitamin D3 deficiency, A/N ratio, degree of AH, season of hospitalization, and the percentages of lymphocytes and basophils (Figure 1). These variables were analyzed using Firth’s penalized logistic regression (Table 3), resulting in four independent risk factors that were statistically significant: preoperative tympanometry results, vitamin D3 deficiency, degree of AH, and age. A predictive model incorporating these four factors was developed and presented as a nomogram (Figure 2). The calibration curve of the nomogram for predicting the risk of COME in children with AH indicated a strong fit between the apparent and ideal lines (Figure 3). The C-index for the prediction nomogram was 0.945 (95% confidence interval [CI] 0.941, 0.949). Bootstrap validation, which recalibrated the coefficients for the final set of predictors, yielded a C-index of 0.934, further confirming the nomogram’s high discriminative power. The incidence of AH with COME in this predictive model was 21.2% (66/311), comparable to the findings of Niedzielski et al. (20.4%, 110/539) [32]. Within this threshold, the decision curve was consistently above the none and all lines (Figure 4), indicating that all patients could benefit from this predictive model within a threshold range of 10%–90%. Thus, this model demonstrates significant clinical applicability.

**Table 2:** Values of continuous variables at specific percentiles

**Variable**	**P5**	**P35**	**P65**	**P95**
Age (year)	3	5	7	11
AH course (month)	4	12	24	60
A/N ratio	0.636	0.760	0.830	0.920
Total IgE	5.485	34.760	99.280	510.600
NEUT%	26.66	38.72	48.70	63.08
LY%	26.50	41.20	49.90	62.34
MONO%	4.00	5.50	6.60	8.62
EOS%	1.1	2.4	4.1	9.5
BASO%	0.2	0.4	0.6	1.0

Abbreviations: BASO%: Percentage of basophils in routine blood examination; EOS%: Percentage of eosinophils in routine blood examination; LY%: Percentage of lymphocytes in routine blood examination; MONO%: Percentage of monocytes in routine blood examination; NEUT%: Percentage of neutrophils in routine blood examination; AH: Adenoid hypertrophy.

**Table 3:** Results of Firth’s penalized logistic regression analysis of potential risk factors for chronic otitis media with effusion (COME) in children with adenoid hypertrophy (AH)

**Variable**	**Comparison**	**Prediction model**		
		**β** ^*^	**Odds ratio (95%CI)**	***P* value**
Age (year)	Age=<3 (Reference)			
	3<Age=<5	--3.880	0.021 (0.002, 0.176)	<0.001
	5<Age=<7	--3.744	0.024 (0.002, 0.183)	<0.001
	7<Age=<11	--4.335	0.013 (0.001, 0.110)	<0.001
	Age>11	--4.643	0.010 (0.000, 0.156)	<0.001
Tympanometry	Normal (Reference)			
	Type C	3.870	47.93 (13.54, 169.74)	<0.001
	Type B	5.004	148.94 (40.54, 547.43)	<0.001
VD3 deficiency	No (Reference)			
	Yes	1.431	4.18 (1.49, 11.75)	0.006
Degree	Moderate (Reference)			
	Pathologic	2.682	14.61 (1.93, 110.40)	0.008
Intercept		--6.731	0.001 (0.000, 0.20)	0.007

^*^β is the regression coefficient

## Discussion

This study developed a noninvasive diagnostic tool for COME in children with AH. Traditional diagnostic methods for COME include physical examinations and tympanometry. Physical examination of the ear is conducted using standard otoscopy or the maneuver method, wherein the presence of fluid in the middle ear is assessed through visual inspection of the tympanic membrane and tympanic cavity. However, electronic otoscopy may induce anxiety in children, leading to crying, which can obscure the examiner’s judgment. Consequently, cases of COME may be missed or misdiagnosed. Watters et al. [16] demonstrated that endoscopy alone is insufficient for reliably detecting middle ear effusion. Additionally, Ma et al. [33] reported that only 63.75% of children with COME exhibited middle ear effusion during examination. In our study, all children underwent a comprehensive physical examination prior to surgery, revealing a diagnostic rate of middle ear effusion of 65.2% (43/66) in the AH with COME group. The missed diagnoses may stem from the subjective nature of otoscopic judgments, which can vary based on examiner experience.

Tympanometry, a noninvasive and objective method for detecting pressure changes in the middle ear, is frequently employed to assist in diagnosing COME. Wu et al. [34] indicated that the preoperative tympanometric B-type curve pattern has a higher diagnostic rate for middle ear effusion. Despite this, traditional otological examination methods still exhibit room for improvement regarding their detection rates for COME. During our investigation, we considered including audiological factors; only auditory brainstem response (ABR) testing can accurately assess hearing loss across all pediatric age groups [10]. However, this diagnostic tool is not readily available in primary healthcare settings in China.

Recent guidelines in various countries have strongly advocated for pneumatic otoscopy as a critical examination method for diagnosing OME [10, 35, 36]. However, since pneumatic otoscopy requires significant cooperation from pediatric patients and has not been widely adopted, the proportion of diagnosed middle ear effusion cases remains low. In primary healthcare, only 7%–33% of physicians utilize pneumatic otoscopy for diagnosing middle ear effusion [10]. Some researchers have reported high specificity and sensitivity for routine middle ear and mastoid scans in diagnosing middle ear effusion [37]; however, due to radiation exposure, these scans are not recommended for younger patients.

In conclusion, the present study developed an accessible noninvasive tool by analyzing independent risk factors for COME in children with AH and evaluating its efficacy and clinical significance. The nomogram integrates traditional otological examinations with easily obtainable clinical data, enabling clinicians to calculate the probability of COME for children with AH based on outpatient examination results.

The risk factors for COME in children with AH have been extensively studied [38]. While the pathogenesis of COME remains unclear, multiple factors contribute to its development. These factors include respiratory tract infections, AH, maxillofacial deformities, allergic and immune responses, family environment, breastfeeding practices, family economic status, and parental education levels [8–10]. However, previous studies have yielded varying results. This study identified independent risk factors for COME in children with AH, including the degree of AH, vitamin D3 deficiency, age, and preoperative tympanometry results.

To assess the severity of AH, the study utilized lateral nasopharyngeal radiographs. Findings indicated that the degree of AH correlates with the development of COME; specifically, more severe AH increases the risk of COME. The Eustachian tube, an essential anatomical structure, regulates air pressure in the middle ear. Hypertrophic adenoid tissue can mechanically obstruct the pharyngeal orifice of the Eustachian tube, leading to negative pressure in the middle ear and the accumulation of effusion [13]. Additionally, hypertrophic adenoids serve as reservoirs for bacteria that form biofilms. Microorganisms from the upper respiratory tract may ascend through the Eustachian tube mucosa to the middle ear, resulting in retrograde infections [39]. Although lateral nasopharyngeal radiographs provide only a two-dimensional assessment of AH and may not fully illustrate the specific anatomical relationship of adenoid tissue obstructing the Eustachian tube’s pharyngeal opening, numerous studies have demonstrated a significant correlation between measurements from lateral radiographs and those obtained via endoscopic evaluation [40, 41]. Thus, lateral nasopharyngeal radiography remains a valuable tool for estimating adenoid size and guiding surgical decisions.

Vitamin D3, the biologically active form of vitamin D in humans, is synthesized in the skin through ultraviolet-induced photoconversion of cholesterol and can also be obtained from dietary sources, such as animal liver. For healthy individuals, sunlight-derived vitamin D3 typically meets physiological requirements. Growing evidence suggests a link between vitamin D3 deficiency and the incidence of COME in children. A randomized controlled trial found that daily supplementation with 1000 IU of vitamin D3 over four months significantly reduced the incidence of OME to 44.8%, compared to 66.5% in the placebo group (*P* ═ 0.03) among 116 children [42]. This finding is further supported by a cohort study conducted by Walker et al. [43], which indicated that higher serum vitamin D3 levels correlate with a decreased risk of COME. Consistent with these reports, the nomogram developed in our study revealed that lower serum vitamin D3 levels are associated with an increased risk of COME in children with AH. Notably, the prevalence of vitamin D3 deficiency was significantly higher in AH patients with COME compared to those without (65.2% vs 24.9%, *P* < 0.01). This deficiency may contribute to pathological changes such as squamous metaplasia of the tympanic mucosa and impaired mucociliary clearance [44], facilitating the persistence of effusion. The pathogenic process likely involves colonized bacteria from hypertrophic adenoids migrating via the Eustachian tube, inducing goblet cell hyperplasia in the middle ear epithelium and leading to mucus-rich effusion with elevated mucin content [45]. Importantly, goblet cell proliferation often persists despite months of treatment, indicating potential irreversibility [46]. Vitamin D3 may modulate this process through various mechanisms, as higher serum levels enhance the expression of cathelicidin, an antimicrobial peptide; however, elevated mucin concentrations in middle ear effusion may compromise its efficacy [47, 48]. From an etiopathological perspective, vitamin D plays a critical role in regulating several key processes implicated in COME, including immune cytokine networks, Th1/Th2 balance, pathogen eradication, and Eustachian tube function [49–52]. Dysregulation of Th1/Th2 immunity, particularly Th2-cell overactivation, is a significant mechanism in the development of COME. Experimental studies have demonstrated that vitamin D3 can suppress T-lymphocyte proliferation and cytokine secretion, thereby mitigating immune-mediated tissue injury, inhibiting inflammatory cell activation, and reducing local mucin production [53, 54]. However, the causal relationship between vitamin D3 deficiency and the development of COME remains unclear. Scholars have suggested that the persistence of inflammatory states may affect vitamin D conversion [55–57]. Consequently, the high prevalence of vitamin D3 deficiency in children with COME may be a result of chronic otitis media.

This study examined the seasonal patterns of hospitalization among children with AH and COME. Notably, 59.1% of children in the AH group with COME were admitted for treatment during the winter and spring months, compared to only 35.5% of those in the AH group without COME. A statistically significant difference in seasonal admissions was observed between the two groups (χ^2^ ═ 4.419, *P* ═ 0.031). However, this characteristic was not significant in LASSO regression analyses, likely due to the correlation between seasonal variations and serum vitamin D3 levels. LASSO regression effectively mitigates multicollinearity among factors by implementing feature selection. During winter and early spring, shorter daylight hours reduce children’s outdoor activities, leading to decreased vitamin D levels, which are closely associated with sunlight exposure and generally diminish during these seasons [45, 58]. Further research is necessary to explore the relationship between the incidence of COME in children with AH and daily sunshine duration.

The nomogram developed in this study indicated that younger children are at a heightened risk for developing COME. Previous studies, both domestic and international, have shown a correlation between younger age groups and the incidence of COME [59, 60]. The youngest patient in the AH with COME group was just 3 years old. Younger children often exhibit limited verbal skills, reduced attention spans, and difficulty understanding illness concepts or distinguishing between physical symptoms. Consequently, parents of young children with AH should remain vigilant regarding the potential for middle ear effusion.

This study introduced a predictive model for COME in patients with AH, utilizing risk factors and tympanometry results presented in a clinically applicable nomogram. This model serves as a personalized and relatively accurate tool for predicting COME, offering significant clinical value given the challenges in early detection of this condition. Specifically, young children with AH frequently struggle to articulate symptoms, and parental awareness of COME is often insufficient [38]. Consequently, medical intervention is typically sought only when children demonstrate substantial hearing loss or behavioral issues, such as concentration difficulties noted by educators. Additionally, children residing in rural areas face a greater risk of undiagnosed hearing impairments compared to their urban peers [61]. Consistent with previous reports, our study found that only 31.7% of patients with AH and COME actively reported hearing loss, a rate aligned with findings by Brennan-Jones et al. [15].

The model exhibited robust discriminative ability, achieving a C-index of 0.934 during internal validation, underscoring its potential for broader application in identifying and managing COME in children with AH. To enhance clinical utility, we established a three-tier risk stratification system based on predicted probabilities: low-risk (<20%), intermediate-risk (20%–60%), and high-risk (>60%). Corresponding management strategies were proposed, including watchful waiting with periodic follow-up for low-risk patients, referral for specialized audiological testing for intermediate-risk cases, and consultation with a specialist for high-risk individuals. This framework aims to serve as a clinical decision support tool, with final treatment tailored to individual patient conditions.

This retrospective study has several limitations. The selected risk factors were derived from prior studies and clinical observations. While existing literature has established associations between family socioeconomic status, indoor air pollution, family size, and pet ownership with the occurrence of COME in children [9, 62, 63], these potential socioeconomic factors were not included in our analysis. Additionally, evidence suggests that COVID-19 and its variants may influence the development and persistence of middle ear effusion [64]. Future studies should investigate the extent to which these factors contribute to the development of COME in children with AH. The bootstrap resampling approach was limited to refitting model coefficients for the final set of predictors without repeated variable screening. Furthermore, this single-center retrospective study necessitates validation through larger external datasets to confirm the accuracy and specificity of the nomogram. Given practical constraints, including limited equipment and technical capabilities in Chinese primary hospitals, this study did not analyze hearing loss in children or employ wideband acoustic immittance (WAI) to assess middle ear effusion. Although existing research has demonstrated that WAI offers higher sensitivity and specificity than conventional 226 Hz acoustic immittance in detecting middle ear effusion [65–67], such methods were not feasible in our setting. Future investigations will aim to incorporate more comprehensive audiological assessments to further explore the mechanistic relationships between COME and AH.

## Conclusion

This study established a supplemental risk prediction model for COME in children with AH, based on preoperative tympanometry, the degree of AH, age, and serum vitamin D3 levels. Clinicians can utilize this nomogram to estimate the probability of this outcome and provide targeted medical interventions, thereby mitigating the adverse effects of COME on the growth and development of children with AH. The findings of this study require validation through external data, and further research is necessary to confirm the clinical significance of this nomogram.

## Data Availability

The data that support the findings of this study are available from the corresponding author upon reasonable request.

## References

[1] Editorial Board of Chinese Journal of Otorhinolaryngology H, Neck S, Subspecialty Group of P (2021). [Guideline for the diagnosis and treatment of otitis media with effusion in children (2021)]. Zhonghua Er Bi Yan Hou Tou Jing Wai Ke Za Zhi.

[2] Baggi E, Semino M, Bianchini S, Fattizzo M, Rosazza C, Esposito S (2013). Middle ear problems in children hospitalised because of lower respiratory tract infections: a comparison between two cohorts in Burundi and Italy. Int J Pediatr Otorhinolaryngol.

[3] Parmar S, Davessar JL, Singh G, Arora N, Kansal L, Singh J (2019). Prevalence of otitis media with effusion in children with hearing loss. Indian J Otolaryngol Head Neck Surg.

[4] Biagio L, Swanepoel DW, Laurent C, Lundberg T (2014). Paediatric otitis media at a primary healthcare clinic in South Africa. S Afr Med J.

[5] Tos M (1984). Epidemiology and natural history of secretory otitis. Am J Otol.

[6] Vanneste P, Page C (2019). Otitis media with effusion in children: pathophysiology, diagnosis, and treatment. A review. J Otol.

[7] Li S, Huang Y, Chen X, Wang W, Zhang Q, Zhang Q (2020). [Effect of otitis media with effusion on vestibular function in children: a pilot study]. Lin Chuang Er Bi Yan Hou Tou Jing Wai Ke Za Zhi.

[8] Aniansson G, Alm B, Andersson B, Hakansson A, Larsson P, Nylen O (1994). A prospective cohort study on breast-feeding and otitis media in Swedish infants. Pediatr Infect Dis J.

[9] Songu M, Islek A, Imre A, Aslan H, Aladag I, Pinar E (2020). Risk factors for otitis media with effusion in children with adenoid hypertrophy. Acta Otorhinolaryngol Ital.

[10] Rosenfeld RM, Shin JJ, Schwartz SR, Coggins R, Gagnon L, Hackell JM (2016). Clinical practice guideline: otitis media with effusion (update). Otolaryngol Head Neck Surg.

[11] Aslıer M, Aslıer NGY, Ercan İ, Keskin S (2022). Clustering upper airway physicals, otitis media with effusion and auditory functions in children. Auris Nasus Larynx.

[12] Li P, Li T, Yu L, Chen A, Wu Y, Wan Y (2025). Predictive value of adenoid-nasopharyngeal ratio in the diagnosis of secretory otitis media. Ear Nose Throat J.

[13] Austin DF (1989). Adenoidectomy for secretory otitis media. Arch Otolaryngol Head Neck Surg.

[14] Gleeson MJ (2008). Scott-Brown’s otorhinolaryngology, head and neck surgery, 7th edition. Proc Prehistor Soc.

[15] Brennan-Jones CG, Whitehouse AJ, Park J, Hegarty M, Jacques A, Eikelboom RH (2015). Prevalence and risk factors for parent-reported recurrent otitis media during early childhood in the Western Australian Pregnancy Cohort (Raine) Study. J Paediatr Child Health.

[16] Watters GW, Jones JE, Freeland AP (1997). The predictive value of tympanometry in the diagnosis of middle ear effusion. Clin Otolaryngol Allied Sci.

[17] Xian ZX, Li L, Liang ZJ (2007). Comparison of the diagnostic value of nasopharynx lateral film and electronic nasopharyngoscope in children with adenoid hypertrophy. J Clin Otorhinol Head Neck Surg.

[18] Pan SL, Zhou F, Zhou YH, Zhong PD (2023). Application of lateral nasopharynx film in diagnosis of adenoid hypertrophy in children in township hospitals. Smart Healthc.

[19] Fujioka M, Young LW, Girdany BR (1979). Radiographic evaluation of adenoidal size in children: adenoidal-nasopharyngeal ratio. AJR Am J Roentgenol.

[20] Alvarez D, Gutierrez-Tobal GC, Del Campo F, Hornero R (2015). Positive airway pressure and electrical stimulation methods for obstructive sleep apnea treatment: a patent review (2005-2014). Expert Opin Ther Pat.

[21] Sharifkashani S, Dabirmoghaddam P, Kheirkhah M, Hosseinzadehnik R (2015). A new clinical scoring system for adenoid hypertrophy in children. Iran J Otorhinolaryngol.

[22] Adedeji TO, Amusa YB, Aremu AA (2016). Correlation between adenoidal nasopharyngeal ratio and symptoms of enlarged adenoids in children with adenoidal hypertrophy. Afr J Paediatr Surg.

[23] Chen WB, Wang YC.

[24] Masna K, Zwierz A, Domagalski K, Burduk P (2021). The impact of the thermal seasons on adenoid size, its mucus coverage and otitis media with effusion: a cohort study. J Clin Med.

[25] Group WHOMGRS (2006). WHO Child Growth Standards based on length/height, weight and age. Acta Paediatr Suppl.

[26] De Onis M, Onyango AW, Borghi E, Siyam A, Nishida C, Siekmann J (2007). Development of a WHO growth reference for school-aged children and adolescents. Bull World Health Organ.

[27] Holick MF, Chen TC (2008). Vitamin D deficiency: a worldwide problem with health consequences. Am J Clin Nutr.

[28] Harrell FE (2010). Regression modeling strategies: with applications to linear models, logistic regression, and survival analysis. Berlin: Springer;.

[29] Sauerbrei W, Royston P, Binder H (2007). Selection of important variables and determination of functional form for continuous predictors in multivariable model building. Stat Med.

[30] Friedman J, Hastie T, Tibshirani R (2010). Regularization paths for generalized linear models via coordinate descent. J Stat Softw.

[31] Heinze G, Schemper M (2002). A solution to the problem of separation in logistic regression. Stat Med.

[32] Niedzielski A, Chmielik LP, Kasprzyk A, Stankiewicz T, Mielnik-Niedzielska G (2021). Health-related quality of life assessed in children with adenoid hypertrophy. Int J Environ Res Public Health.

[33] Ma R, Tian L, Ren DD (2013). Clinical diagnosis and audiological characteristics of otitis media with effusion in elder children combined with adenoid hypertrophy. Chin J Ophthalmol Otorhinolaryngol.

[34] Wu S, Zhao W, Mai QW, Gao MQ, Lin QW, Zheng YG (2019). Comparison of the clinical value and the differential diagnostic of otitis media with effusion between 226 Hz, 1000 Hz acoustic immittance and wideband immittance. Chinese J Ophthalmol Otorhinolaryngol.

[35] Hidaka H, Ito M, Ikeda R, Kamide Y, Kuroki H, Nakano A (2023). Clinical practice guidelines for the diagnosis and management of otitis media with effusion (OME) in children in Japan—2022 update. Auris Nasus Larynx.

[36] Blanc F, Ayache D, Calmels MN, Deguine O, Francois M, Leboulanger N (2018). Management of otitis media with effusion in children. Societe francaise d’ORL et de chirurgie cervico-faciale clinical practice guidelines. Eur Ann Otorhinolaryngol Head Neck Dis.

[37] Ren DD, Wang WQ (2012). Assessment of middle ear effusion and audiological characteristics in young children with adenoid hypertrophy. Chin Med J (Engl).

[38] Veivers D, Williams GM, Toelle BG, Waterman AMC, Guo Y, Denison L (2022). The indoor environment and otitis media among Australian children: a national cross-sectional study. Int J Environ Res Public Health.

[39] Ricci A, Avanzini MA, Scaramuzza C, Castellazzi AM, Marconi M, Marseglia GL (2005). Toll-like receptor 2-positive and Toll-like receptor 4-positive cells in adenoids of children exposed to passive smoking. J Allergy Clin Immunol.

[40] Wang M, Jing H, Guo H, Li X, Sun J (2022). [Evaluation of Eustachian tube function in children with adenoid hypertrophy by nasopharyngeal digital photography and ETDQ-7 scores]. Lin Chuang Er Bi Yan Hou Tou Jing Wai Ke Za Zhi.

[41] Maw AR, Jeans WD, Fernando DC (1981). Inter-observer variability in the clinical and radiological assessment of adenoid size, and the correlation with adenoid volume. Clin Otolaryngol Allied Sci.

[42] Marchisio P, Consonni D, Baggi E, Zampiero A, Bianchini S, Terranova L (2013). Vitamin D supplementation reduces the risk of acute otitis media in otitis-prone children. Pediatr Infect Dis J.

[43] Walker RE, Bartley J, Camargo CA Jr, Flint D, Thompson JMD, Mitchell EA (2017). Higher serum 25(OH)D concentration is associated with lower risk of chronic otitis media with effusion: a case-control study. Acta Paediatr.

[44] Akcan FA, Dundar Y, Akcan HB, Uluat A, Cebeci D, Unlu I (2019). Evaluation of nasal mucociliary clearance time in patients with Vitamin-D deficiency. Eur Arch Otorhinolaryngol.

[45] Walker RE, Bartley J, Camargo CA Jr, Mitchell EA (2019). Vitamin D and otitis media. Curr Allergy Asthma Rep.

[46] Caye-Thomasen P, Tos M (2002). Histopathologic differences due to bacterial species in acute otitis media. Int J Pediatr Otorhinolaryngol.

[47] Ooi JH, Li Y, Rogers CJ, Cantorna MT (2013). Vitamin D regulates the gut microbiome and protects mice from dextran sodium sulfate-induced colitis. J Nutr.

[48] Felgentreff K, Beisswenger C, Griese M, Gulder T, Bringmann G, Bals R (2006). The antimicrobial peptide cathelicidin interacts with airway mucus. Peptides.

[49] Li DP, Huang H, He M (2017). Recent progress in diagnosis and treatment of secretory otitis media. Chin J Otol.

[50] Sadeghi K, Wessner B, Laggner U, Ploder M, Tamandl D, Friedl J (2010). Vitamin D3 down-regulates monocyte TLR expression and triggers hyporesponsiveness to pathogen-associated molecular patterns. Eur J Immunol.

[51] Li Y, Zhao SQ (2016). Advances in clinical research on otitis media with effusion and allergic rhinitis in children. Chin J Otol.

[52] Aryan Z, Rezaei N, Camargo CA Jr (2017). Vitamin D status, aeroallergen sensitization, and allergic rhinitis: a systematic review and meta-analysis. Int Rev Immunol.

[53] Khoo AL, Joosten I, Michels M, Woestenenk R, Preijers F, He XH (2011). 1,25-Dihydroxyvitamin D3 inhibits proliferation but not the suppressive function of regulatory T cells in the absence of antigen-presenting cells. Immunology.

[54] Moore KW, De Waal Malefyt R, Coffman RL, O’garra A (2001). Interleukin-10 and the interleukin-10 receptor. Annu Rev Immunol.

[55] Mangin M, Sinha R, Fincher K (2014). Inflammation and vitamin D: the infection connection. Inflamm Res.

[56] Waldron JL, Ashby HL, Cornes MP, Bechervaise J, Razavi C, Thomas OL (2013). Vitamin D: a negative acute phase reactant. J Clin Pathol.

[57] Custodio G, Schwarz P, Crispim D, Moraes RB, Czepielewski M, Leitao CB (2018). Association between vitamin D levels and inflammatory activity in brain death: a prospective study. Transpl Immunol.

[58] Giustina A, Lazaretti-Castro M, Martineau AR, Mason RS, Rosen CJ, Schoenmakers I (2024). A view on vitamin D: a pleiotropic factor?. Nat Rev Endocrinol.

[59] Norhafizah S, Salina H, Goh BS (2020). Prevalence of allergic rhinitis in children with otitis media with effusion. Eur Ann Allergy Clin Immunol.

[60] Caylan R, Bektas D, Atalay C, Korkmaz O (2006). Prevalence and risk factors of otitis media with effusion in Trabzon, a city in northeastern Turkey, with an emphasis on the recommendation of OME screening. Eur Arch Otorhinolaryngol.

[61] Luo Y, He P, Wen X, Gong R, Hu X, Zheng X (2022). Otitis media and its association with hearing loss in Chinese adults: a population based study of 4 provinces in China. Front Public Health.

[62] Bergroth E, Remes S, Pekkanen J, Kauppila T, Buchele G, Keski-Nisula L (2012). Respiratory tract illnesses during the first year of life: effect of dog and cat contacts. Pediatrics.

[63] Da Costa JL, Navarro A, Neves JB, Martin M (2004). Household wood and charcoal smoke increases risk of otitis media in childhood in Maputo. Int J Epidemiol.

[64] Meng X, Wang Y, Han C, Gu X, Hang C, Guo J (2024). Clinical manifestations and outcomes of otitis media with effusion in adult patients following Omicron infection in China. Biomol Biomed.

[65] Keefe DH, Sanford CA, Ellison JC, Fitzpatrick DF, Gorga MP (2015). Wideband aural acoustic absorbance predicts conductive hearing loss in children. Int J Audiol.

[66] Ellison JC, Gorga M, Cohn E, Fitzpatrick D, Sanford CA, Keefe DH (2012). Wideband acoustic transfer functions predict middle-ear effusion. Laryngoscope.

[67] Hunter LL, Feeney MP, Miller JaL, Jeng PS, Bohning SJE (2010). Wideband reflectance in newborns: normative regions and relationship to hearing-screening results. Ear Hear.

